# Multiple Targets of the Canonical WNT/β-Catenin Signaling in Cancers

**DOI:** 10.3389/fonc.2019.01248

**Published:** 2019-11-18

**Authors:** Yves Lecarpentier, Olivier Schussler, Jean-Louis Hébert, Alexandre Vallée

**Affiliations:** ^1^Centre de Recherche Clinique, Grand Hôpital de l'Est Francilien, Meaux, France; ^2^Research Laboratory, Department of Cardiovascular Surgery, Geneva University Hospitals, Geneva, Switzerland; ^3^Institut de Cardiologie, Hôpital de la Pitié-Salpétrière, Paris, France; ^4^Hypertension and Cardiovascular Prevention Unit, Diagnosis and Therapeutic Center, Hôtel-Dieu Hospital, AP-HP, Paris, France; ^5^DACTIM-MIS, LMA, UMR CNRS 7348, CHU de Poitiers, Université de Poitiers, Poitiers, France

**Keywords:** canonical WNT/β-catenin signaling, immune cycle, cell division cycle, myofibroblast, circadian rhythms, Warburg glycolysis, TGF-β1, immunotherapy

## Abstract

Canonical WNT/β-catenin signaling is involved in most of the mechanisms that lead to the formation and development of cancer cells. It plays a central role in three cyclic processes, which are the cell division cycle, the immune cycle, and circadian rhythms. When the canonical WNT pathway is upregulated as in cancers, the increase in β-catenin in the nucleus leads to activation of the expression of numerous genes, in particular CYCLIN D1 and cMYC, where the former influences the G1 phase of the cell division cycle, and the latter, the S phase. Every stage of the immune cycle is disrupted by the canonical WNT signaling. In numerous cancers, the dysfunction of the canonical WNT pathway is accompanied by alterations of the circadian genes (CLOCK, BMAL1, PER). Induction of these cyclic phenomena leads to the genesis of thermodynamic mechanisms that operate far from equilibrium, and that have been called “dissipative structures.” Moreover, upregulation of the canonical WNT/β-catenin signaling is important in the myofibroblasts of the cancer stroma. Their differentiation is controlled by the canonical WNT /TGF-β1 signaling. Myofibroblasts present ultraslow contractile properties due to the presence of the non-muscle myosin IIA. Myofibroblats also play a role in the inflammatory processes, often found in cancers and fibrosis processes. Finally, upregulated canonical WNT deviates mitochondrial oxidative phosphorylation toward the Warburg glycolysis metabolism, which is characteristic of cancers. Among all these cancer-generating mechanisms, the upregulated canonical WNT pathway would appear to offer the best hope as a therapeutic target, particularly in the field of immunotherapy.

## Introduction

**Figure 1:**
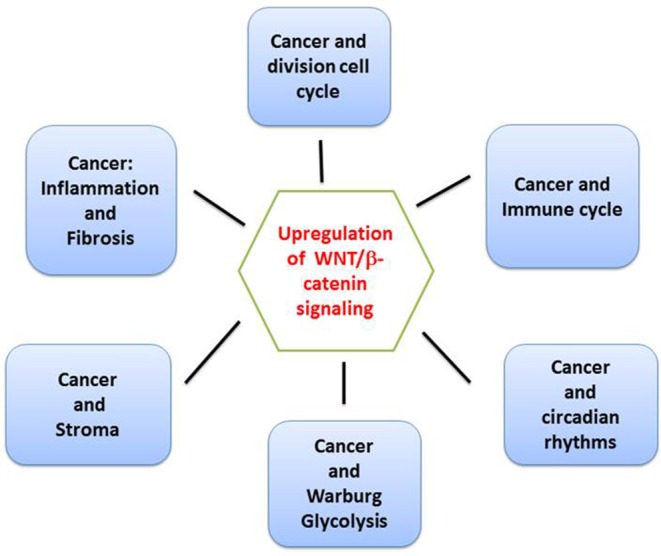
Overview of checkpoints of the canonical WNT/β-catenin signaling. The canonical WNT signaling is involved in at least six key areas of cancer pathophysiology, i.e., cell division cycle, immune cycle, circadian rhythms, cancer stroma, Warburg glycolysis, inflammation, and fibrosis.

Since the discovery in 1982 of Int1 (WNT1a) as an oncogene reported in murine breast cancers, the canonical WNT/β-catenin pathway has often been found to be associated with cancer (1). Indeed, the canonical WNT pathway represents a key element in the genesis and development of a large number of human cancers. This makes the pathway a prime target in the search for new treatments for cancers, with high mortality and morbidity. In this review, we summarize the main findings regarding the dysfunctions of the canonical WNT signaling encountered in many cancers (Figure 1). It has been reported that three major cyclic processes namely the cell division cycle (2–4), the immune cycle (5), and circadian rhythms (CRs) (6) become highly disturbed due to upregulation of the canonical WNT signaling. In the division cell cycle, canonical WNT signaling was initially described as a trigger for the G1 phase advancement through CYCLIN D1 and c-MYC transcription (7–11). This signaling is also involved at every stage of the immune cycle (12) and has led to the development of new modalities of immunotherapy. In addition, the dysfunction of the canonical WNT signaling is involved in the generation and development of the cancer stroma (13, 14). In particular, aerobic glycolysis in tumor cells, which is partly responsible for shunting the mitochondrial oxidative phosphorylations [Warburg effect (15)], is related to the upregulation of the canonical WNT pathway. Finally, cancers fit into the thermodynamic framework of dissipative structures, in a manner similar to CRs or by generating oscillating phenomena in which canonical WNT pathway plays a central role (16). Since β-catenin regulates the expression of a considerable number of genes, such as CYCLIN D1 and cMYC, it is not surprising that this signaling interferes in numerous cancer-generating processes. Other pathways such as TGF-β1, activate the canonical WNT signaling, and will therefore potentially play a role upstream.

## General Remarks About the Canonical WNT/β-Catenin Signaling

**Figure 2:**
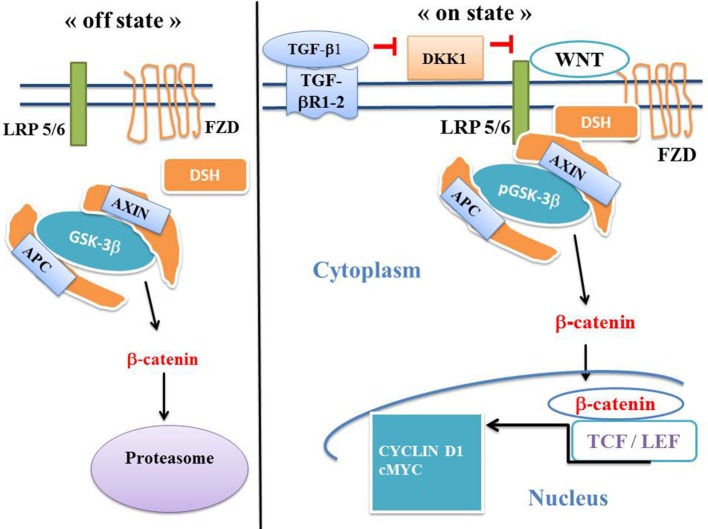
The canonical WNT/β-catenin pathway: “on” and “off” states. The canonical WNT pathway can be in either “on-state” or “off-state.” In the “off state”, i.e., in the absence of WNT ligand or in the presence of the active form of the glycogen synthase kinase-3β (GSK-3β) (i.e., the unphosphorylated form GSK-3β), cytosolic β-catenin is phosphorylated by GSK-3β and undergoes the destruction process into the proteasome. In the “on-state,” a WNT ligand binds both FZD and LRP5/6 receptors. GSK-3β is then under the inactive form (i.e., the phosphorylated form pGSK-3β). This leads to activation of the phosphoprotein Disheveled (DSH). DSH recruits the destruction complex (pGSK-3β + AXIN +APC) to the plasma membrane, where AXIN directly binds the cytoplasmic tail of LRP5/6. APC is the adenomatous polyposis coli. Activation of DSH leads to the inhibition of both phosphorylation and degradation of β-catenin. β-catenin accumulates into the cytosol and then translocates to the nucleus to bind the LEF-TCF co-transcription factors. This induces the WNT-response gene transcription of numerous genes such as *CYCLIN D1, cMYC, MCT1, PDK, fibronectin*. DKK1 inhibits the canonical WNT signaling.

The canonical WNT signaling is involved in numerous physiological processes such as embryonic development, metabolism, cell fate and epithelial-mesenchymal transition (EMT). Upregulation of the canonical WNT pathway increases the level of β-catenin in both the cytoplasm and nucleus. The dysfunction of this pathway is involved in numerous pathological processes, particularly in cancers and fibrosis (17–20). The T-cell /lymphoid enhancer (TCF/LEF) transcription factors represent the final effectors of the canonical WNT signaling (Figure 2). The destruction complex is composed of AXIN, tumor suppressor adenomatous polyposis coli (APC), and glycogen synthase kinase-3 (GSK-3β). GSK-3β exerts a tight control on the canonical WNT pathway. When WNT ligands are absent (“off state”), the cytosolic β-catenin is continuously ubiquitinylated leading to degradation via sequential phosphorylations by GSK-3β and caseine kinase 1 (CK1). The destruction complex phosphorylates β-catenin. Then β-catenin is degraded within the proteasome. When WNT ligands are present (“on state”), the WNT receptor interacts with Frizzled (FZL) and LDL receptor-related protein 5/6 (LRP5/6). WNT receptor is linked with Disheveled (DSH). This favors the disruption of the destruction complex and hinders degradation of β-catenin in the proteasome. β-catenin translocates to the nucleus and interferes with the TCF- LEF co-transcription factors. This leads to the stimulation of several β-catenin target genes (CYCLIN D1, c-MYC, PDK, MTC-1, MMP7, fibronectin, COX-2, AXIN-2) (7, 8, 21, 22) (Figure 2). Mutations in the β-catenin gene (CTNNB1), mutations or loss-of-function in APC, AXIN-1 and AXIN-2 genes, all induce a continuous stimulation of the β-catenin signaling and have been reported in a large number of human cancers.

**Figure 3:**
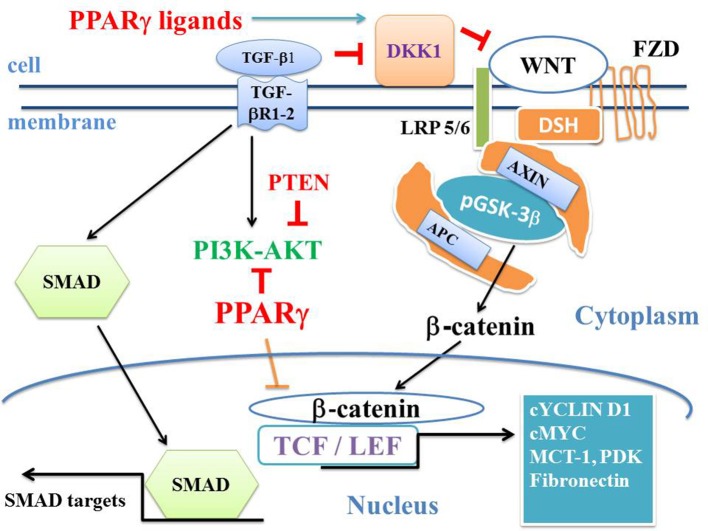
WNT/β-catenin, TGF-β1, PI3K-AKT, SMAD, and PPARγ pathways. “On state” canonical WNT: In the presence of WNT ligands, the WNT receptor binds LRP5/6 and FZD receptors and initiates LRP phosphorylation and DSH-mediated Frizzled internalization. Then, the pGSK-3β/AXIN/APC destruction complex is dissociated. Phosphorylation of β-catenin is inhibited and β-catenin increases in the cytosol and then translocates to the nucleus. β-catenin binds the TCF-LEF transcription factors and induces the WNT-response gene transcription (CYCLIN D1, cMYC, PDK, MCT-1, AXIN2, fibronectin). DKK1 is activated by PPARγ and inhibits WNT. TGF-β1 binds the TGF-βR2 receptor, which recruits the TGF-βR1 receptor. This leads to the formation of a heterotetramer that phosphorylates SMAD. The SMAD complex then translocates to the nucleus and regulates the transcription of Smad target genes (CTGF, COL1A). PI3K-AKT pathway is a non-SMAD signaling and inactivates GSK-3β. PTEN inhibits PI3K-AKT and PPARγ inhibits AKT. PPARγ inhibits the β-catenin/TCF-LEF-induced activation of WNT target genes. TGF-β1 enhances the canonical WNT pathway through the inhibition of DKK1, itself an inhibitor of the canonical WNT signaling.

There is an opposed link between the canonical WNT pathway and PPARγ (23) (Figure 3). This has been established in several cancers, neuro-degenerative diseases, cardiovascular diseases, fibrosis, and numerous other pathological processes (24–32). Transforming growth factor1 (TGF-β1) acts as a driver of these two major pathways (33). TGF-β1 and canonical WNT signaling are closely related, whereby the former activates the later. TGF-β1 receptors are the transmembrane proteins Type I (TGFβRI) and Type II (TGFβRII) (Figure 3). Moreover, TGF-β1 activates both SMAD and non-SMAD pathways such as PI3K-AKT, MAPK and RHO, and downregulates PPARγ expression through the SMAD pathway (34). TGF-β1 and consequently the canonical WNT pathway play a central role in both cancers and fibrosis. In alveolar epithelial cells (AEC), TGF-β1 induces EMT and leads to lung fibrosis. In idiopathic pulmonary fibrosis, myofibroblasts induce lung fibrosis (35–37). In fibrotic lungs, TGF-β1-induced EMT in type II AEC results in differentiation of fibroblasts into myofibroblasts (38–42).

## Canonical WNT/β-Catenin Signaling, Cell Division Cycle and Cancer

### The Cell Division Cycle

**Figure 4:**
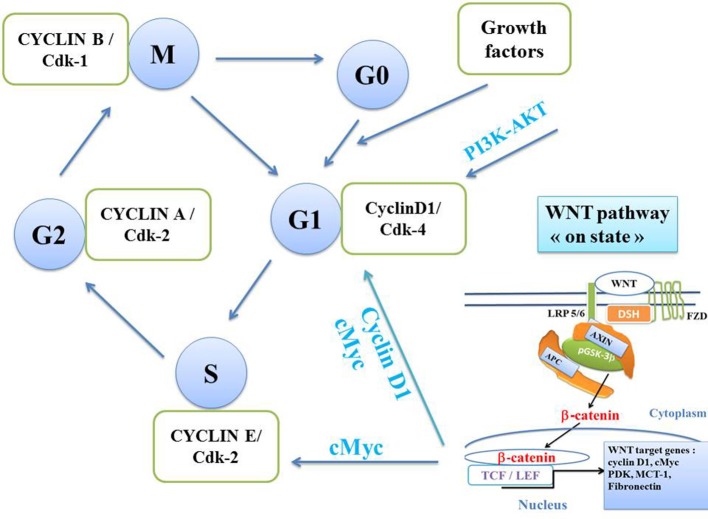
Cell division cycle. Four CYCLIN/Cdk kinase complexes control transitions between the 4 phases of the cell cycle, namely, M, G1, S (DNA replication), and G2 (separating the S phase from the M phase of mitosis). Each phase is controlled by a given CYCLIN/Cdk complex. The transition to a later phase of the cycle can develop only if the previous phase has been totally completed. The cell remains in the quiescent phase (Go) from which it exits when the level of growth factors becomes sufficient and then enters the G1 phase. The G1 phase is controlled by the CYCLIN D1/Cdk4 complex. Phase S is controlled by the CYCLIN E/Cdk2 complex. The G2 phase is controlled by the CYCLIN A/Cdk2 complex. The M phase is controlled by the cyclin B/Cdk1 complex. The canonical WNT signaling increases the expression of *CYCLIN D1* and *cMYC*. CYCLIN D1 induces the G1 phase whereas cMYC induces the S phase.

The canonical WNT signaling plays a central role in the cell division cycle, particularly because the two nuclear β-catenin targets CYCLIN D1 and cMYC intervene at two key checkpoints of the cell cycle. CYCLIN D1 and cMYC act on the G1 phase and cMYC on the S phase (Figure 4). An oscillator rotates the cell cycle, which consists of replicating the DNA. This then leads to a division into two cells, each containing a set of chromosomes (mitosis). In cancer cells, the regulation of the cell division cycle is impaired. Nurse, Hunt, and Hartwell have identified two factors that play an important role in mitosis, namely cyclins and cyclin-dependent kinases (Cdk) that control the transitions between the M, G1, S, and G2 phases of the cell division cycle (4). The key process is a feedback mechanism in which cyclins play a driving role in the cell division cycle (2, 3). Each phase is controlled by a given CYCLIN/Cdk complex. The transition to a later phase of the cycle can only be made if the previous phase has been correctly completed. The cell can remain in the quiescent phase (Go) from which it exits when the level of growth factors is high enough and then allows the onset of the G1 phase. CYCLIN D1 is a key regulator of the checkpoint allowing the G1-S transition, via phosphorylation of the retinoblastoma protein (pRB) complex, thus increasing the cyclin E level. The transcription factor EF2 promotes the cell division cycle and the tumor suppressor pRB slows it down. Cdk kinases are activated by dephosphorylation (cdc25 phosphatase) and inhibited by phosphorylation (Wee1 kinase). The activation of each phase of the cell cycle is sequential and leads to inactivation of the previous phases.

### WNT Transcriptional and Non-transcriptional Effects During the G1 Phase

Several pathways that regulate the cell proliferation act through the G1 phase (43). CYCLIN D1 is inhibited by p21 and p27, allowing the entry of the cell into the quiescence phase Go. The canonical WNT signaling triggers the G1 phase advancement through CYCLIN D1 and c-MYC transcriptional processes (Figures 2, 4) (7–11). The canonical WNT pathway operates through the G1 phase by inducing the transcription of CYCLIN D1 and cMYC which are involved in cell proliferation (10, 44–46). CYCLIN D1 has been one of the first transcriptional target genes of β-catenin to be reported in the colorectal cancer. The transcription factor c-MYC has a dual role in G1 phase by promoting CYCLIN D1 (47) and repressing p21 and p27 (45). pGSK-3β phosphorylates CYCLIN D1, CYCLIN E1, and c-MYC which are direct regulators of G1 progression (48–50).The phosphatidylinositol 3-kinase (PI3K)-protein kinase B (AKT) pathway (PI3K-AKT) allows the G1-S transition phase of the cell cycle through the phosphorylation of GSK-3β (Figure 4). GSK-3β phosphorylation (i.e., pGSK-3β is the inactivated form) decreases the degradation of β-catenin within the proteasome. Thus, TCF/LEF transcription factors are activated, which in turn leads to transcription of the target gene CYCLIN D1 (51). The phosphatase and tensin homolog (PTEM) also decreases cancer cell proliferation due the arrest of the cell cycle in G1 phase (Figures 2, **6**).

### Canonical WNT/β-Catenin Pathway, CYCLIN D1, and Cancer

CYCLIN D1 is considered as key target of the canonical WNT pathway. In endothelial cells, inorganic polyphosphate induces CYCLIN D1 synthesis via activation of the mTOR/WNT/β-catenin pathway (52). CYCLIN D1 is critical for intestinal adenoma development (53). The expression of both CYCLIN D1 and β-catenin, and the inactive pGSK-3β are significantly increased in papillary thyroid carcinoma primary tumors. Expressions of β-catenin and CYCLN D1 are correlated suggesting that β-catenin is a factor driving high CYCLIN D1 levels (54).

### WNT/β-Catenin Signaling, Thermodynamics, Oscillations, and Dissipative Structures

A complete oscillating model of cyclins and cyclin-dependent kinases has been proposed by Gérard and Goldbeter (55). In the presence of a sufficient level of growth factors, the CYCLIN/Cdk complexes are activated sequentially. This complex system is capable of self-organization over time (16, 55, 56). The canonical WNT pathway modulates the cell cycle and conversely the cell cycle influences it. During the cell cycle, the expression of β-catenin oscillates and the maximum activation of β-catenin occurs at the G2/M transition (Figure 4) (57, 58). Moreover, expression of the β-catenin target AXIN2 also peaks at the G2/M transition (59). This requires the LRP6 phosphorylation by CYCLIN Y which mediates phosphorylation of LRP6. The CYCLIN D1/Cdk4 complex peaks at G1. The CYCLIN E/Cdk2 complex peaks at S (Figure 4). The CYCLIN A/Cdk2 complex peaks at the end of S. The peaks of CYCLINS D1, E, and A follow each other, leading to the peak of CYCLIN B. The CYCLIN B/Cdk1 complex peaks at M and triggers the cell division (Figure 4). The sustained oscillations of the various CYCLIN/Cdk complexes reflect the spontaneous self-organization of the cell division cycle over time. Thermodynamically, the cell division cycle represents a dissipative structure which behaves far from equilibrium (60, 61). When a CYCLIN/Cdk complex is graphically represented in an X-Y manner as a function of another complex or as a function of the transcription factor E2F that induces the CYCLIN synthesis, there is an evolution toward a “limit cycle” regardless of initial conditions (55). Below a critical value of growth factor amount corresponding to a bifurcation point, the cell remains in a stable stationary state (quiescent phase Go). Beyond this point, the stationary system becomes unstable and the network of CYCLIN/Cdk complexes begins to oscillate autonomously. Cell proliferation persists as long as the concentration levels of growth factors allow. When the balance between the transcription factor E2F and the suppressor pRB tilts toward a certain level, sustained oscillations appear and the cell division cycle operates. However, even in the absence of growth factor, and under particular conditions, the cell cycle can start beyond a critical point in G1 (“Pardee restriction point”) (62). It must be mentioned that the canonical WNT pathway is involved in another important physiological process. Due to a periodic signal emitted by the segmentation clock, the WNT, NOTCH, and FGF oscillator synchronizes the activation of segmentation genes in successive steps. This leads to the formation of somites during the vertebrate segmentation (63, 64). This represents a supplementary oscillatory dissipative structure controlled by the WNT pathway.

## Canonical WNT/β-Catenin Signaling, Immune Cycle and Cancer

**Figure 5:**
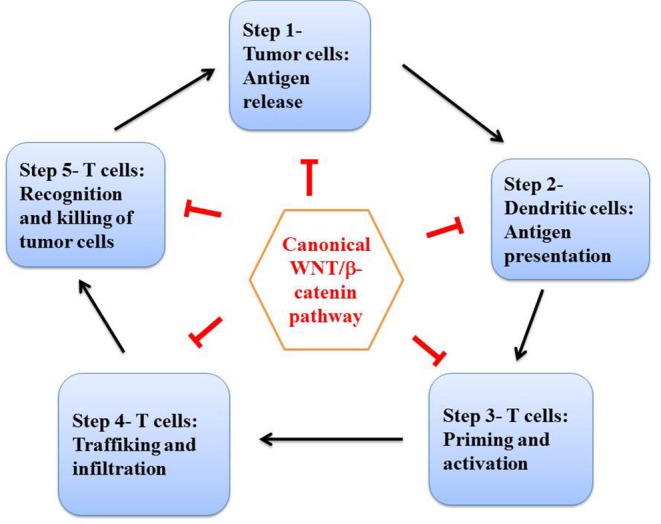
The cancer immune cycle. Adapted and simplified schema of the tumor immune cycle proposed by Chen and Mellman (5) and by Wang et al. (12). Numerous studies have shown that the canonical WNT signaling is involved at each step of the tumor immune cycle: step 1 (tumor antigene release), step 2 (antigene presentation), step 3 (priming and activation), and steps 4 and 5 (trafficking, infiltration, and tumor cell elimination).

The immune cycle (Figure 5) is defined by natural homeostatic oscillations of the immune system when chronic inflammation is occurring such as in cancers, and typically repeated every 7 days. This is due to the synchronous division of T cells over time. T-effector cells boost the immune activity. This is followed by T-regulatory cells that suppress the immune response (65). The cancer-immune cycle has been described by Chen and Mellman (5). The effects of the canonical WNT signaling on the immune cycle have been recently reported in an extensive review by Wang et al. (12). The canonical WNT signaling impacts all the steps of the immune cycle (Figure 4), and we summarize here some of the numerous works concerning this subject. The canonical WNT pathway is a well-recognized cancer promoter as it regulates the cancer immune cycle at most of the checkpoints, composed of dendritic cells, T cells (especially effector T cells, T helper cells, and regulatory T cells) and tumor cells. The treatment of cancers by immunotherapy is being revolutionized by activating or reactivating the tumor-immune cycle (5).

Trafficking of T cells and their infiltration into tumor tissues lead to the antigen release and elimination of cancer cells (66), following a self-propagating cyclic process the final aim of which is to kill cancer cells. Negative regulators of the tumor-immune cycle by using antibodies against cytotoxic T lymphocyte-associated antigen-4 (CTLA-4) and programmed cell death protein1 (PD-1) (67) ameliorate survival rates of patients suffering from metastatic melanoma (68). The canonical WNT signaling is responsible for the onset and advancement of many cancers and is a well-established molecular target for cancer therapy (69–71). Numerous mutations in genes encoding the canonical WNT pathway have been reported in colorectal cancers presenting mutations of APC and β-catenin genes (69, 70, 72). Dysfunction of the canonical WNT signaling has been demonstrated in tumor immunology (73, 74). TCF1 is the endpoint of the canonical WNT signaling (Figure 2) and is a T lymphocyte-specific transcription factor which plays an important role in the development of T cells (75). TCF1 is involved in maturation and secondary expansion of memory CD8^+^ T cells and activates CD8^+^ effector T cells (76).

In cancers, the immune contexture that is partly characterized by the functional orientation of tumor-infiltrating immune cells influences the outcome of patients. Moreover, in metastatic sites, it reflects the corresponding primary cancer (77). The immune-suppressive mesenchymal stem cells (MSCs) have been shown to be an important source of myofibroblasts *in vivo*. We have recently reported *in vitro* that the immune-suppressive capabilities of MSCs are not altered after their differentiation into myofibroblasts (78). In MSCs, involvement of the canonical WNT signaling promotes metastatic growth and chemo-resistance of cholangiocarcinoma (79).

### WNT/β-Catenin Signaling and Dendritic Cells (DCs)

DCs have tumor antigens on the major histocompatibility complex molecules and prime effector T cells. Antigens are released from cancer cells before encountering DCs, then priming and activation of CD4^+^ and CD8^+^ T cells follow. Before priming effector T cells, DCs differentiate into CD103^+^ DCs that are important for recruitment of effector T cells into tumors (80, 81). Activating the mutated β-catenin pathway initiates the gene expression of interferon regulatory factor 8 (IRF8) that leads to differentiation and expansion of CD103^+^ DCs (82). Moreover, activation of β-catenin releases CXCL9/10 in CD103^+^ DCs and inhibits infiltration of effector T cells (81).

### WNT/β-Catenin Signaling and CD8^+^ T Cells

In the tumor-immune cycle, peripheral naïve CD8^+^ T cells differentiate into effector T cells and destroy cancer cells rapidly (81). CD8^+^ T cells are activated and primed by DCs, and then infiltrate tumors to kill cancer cells (83). During tumor development, cancer cells avoid action of the immune cycle by inhibiting CD8^+^ T cell infiltration (84). Mature naïve CD8^+^ T cells are activated by APC and then proliferate in spleen and lymph nodes (5). Upregulation of the WNT/β-catenin pathway induces apoptosis of mature naïve CD8^+^ T cells partially through to the target gene c*-*MYC (85).

### WNT/β-Catenin Pathway and T Helper (Th) Cells

Th cells are subdivided into Th1, Th2, and Th17 and they help the CD8^+^ T cell anti-tumor response through T cell cytokine release (86). Th cells inhibit cancer cells by activating CD27, CD137, and 4-1BB as well as favoring the synthesis of memory CD8^+^ T cells (87). The WNT/β-catenin signaling plays a role in the development and function of Th cells (88–90). It also inhibits Th cells and impairs the anti-tumor immunity. Abnormal WNT/β-catenin pathway impairs the immunity induced by CD4+ T cells. Auto-immune encephalomyelitis activates the canonical WNT pathway that impairs infiltration of CD4^+^ T cells. This can be restored by inhibiting the WNT/β-catenin signaling (90). β-catenin is upregulated in colorectal tumor, and the immunity induced by intra-tumoral CD4^+^ T cells is decreased (89).

### WNT/β-Catenin Signaling and Regulatory T (Treg) Cells

Treg cells support immunosuppression and tolerance to immune stress by inhibiting the effector T (Teff) cell response (91, 92). The canonical WNT signaling is critical for the Treg cell development and exerts a negative regulation on CD4^+^ and CD8^+^ T cells. Infiltration of Treg cells into tumors might be due to the inhibition of the anti-tumor immune response. In cancer, down-regulation of the protein kinase CK2, a negative regulator of canonical WNT pathway, inhibits the Treg-mediated immune suppression (93, 94).

### WNT/β-Catenin Pathway and Tumor Cells

At the last step of the immune cycle, cancer cells are recognized and destroyed by effector T cells. To escape an immune effect, tumor cells express a negative regulatory molecule PD-L1 and generate mutant tumor antigens (95, 96). cMYC, a target of the canonical WNT pathway binds the promoters of the CD47 and PD-L1 genes. cMYC inhibition ameliorates the anti-tumor response (97). Upregulation of the WNT/β-catenin signaling in tumor cells induces T cell exclusion and resistance to immunotherapy. Blockade of the canonical WNT signaling decreases the expression of both CD47 and PD-L1 and improves the anti-tumor immune response (85). In transgenic mouse melanoma, β-catenin stabilization results in a decrease of both T-cell priming and CD8^+^ T cell infiltration (80). Cancer cells remain in a quiescent state after inhibition of the canonical WNT signaling by DKK1 (98). Although cancer immunotherapy by blocking cancer driver genes can benefit patients (99–102), there are several types of resistance to cancer immunotherapy (primary, adaptative, and acquired) (84). Targeting the canonical WNT pathway could help to overcome the immune-resistance.

## WNT/β-Catenin and TGF-β1 Pathways, Tumor Stroma and Myofibroblasts

**Figure 6:**
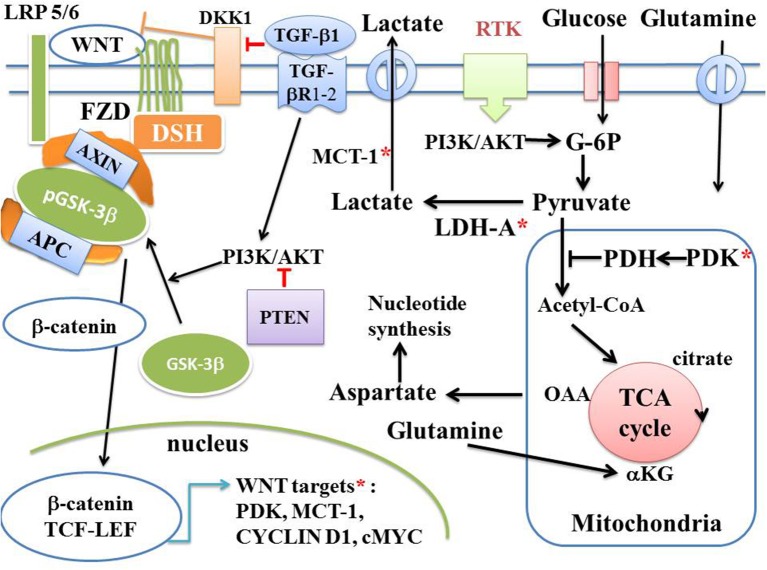
Canonical WNT/β-catenin pathway, Warburg glycolysis, and cancer. Schema of the canonical WNT pathway under aerobic glycolysis in cancer. In the presence of WNT ligands (“on state”), β-catenin accumulates in the cytosol and then translocates to the nucleus to bind TCF-LEF co-transcription factors. This induces the WNT-response gene transcription (CYCLIN D1, cMYC, PDK, MCT-1, fibronectin, etc). Glucose itself activates the canonical WNT pathway. PDK inhibits the PDH complex in mitochondria. Thus, pyruvate cannot be fully converted into acetyl-CoA and cannot enter the TCA cycle. cMYC activates LDH-A which converts cytosolic pyruvate into lactate. MCT-1 favors lactate extrusion out of the cytosol which favors angiogenesis. cMYC increases glutamine entry into both the cytosol and mitochondria. cMYC-induced glutamine enhances aspartate and nucleotide synthesis.

The canonical WNT pathway is tightly involved in the development and maintenance of the tumor stroma. The transforming growth factor-β1 (TGF-β1) and myofibroblasts (103) play a key role in this process. TGF-β1 activates the canonical WNT signaling (Figures 3, 6) and induces the differentiation of fibroblasts into myofibroblasts (31, 33). Myofibroblasts represent key constituents of the tumor stroma which is active in the cancer development as well as in maintaining the tissue architecture. In cancer stroma, myofibroblasts represent the predominant sub-population of cancer-associated cells (104, 105). Myofibroblasts, epithelial cells and connective tissue cells provide favorable conditions for tumor invasion, with loss of epithelial properties and transition to epithelial-mesenchymal characteristics (EMT) (106) that greatly influences the invasive carcinoma advancement and in which the canonical WNT signaling plays a crucial role.

In activated cancer stroma cells, there are neo-formations of contractile stress fibers containing α-smooth muscle actin (α-SMA) and non-muscle myosin (NMMIIA). These cells are named myofibroblasts. In myofibroblasts, there is co-existence of fibroblastic morphological characteristics, such as a well-developed endoplasmic reticulum and α-SMA forming contractile actin microfilament bundles. Proto-myofibroblasts are low contractile cells and when mechanical stress and TGF-β1 are present, they can differentiate into myofibroblasts that express α-SMA and NMMIIA, which are incorporated in stress fibers. This renders myofibroblasts highly contractile (13, 107).

In cancers, mechanical and chemical conditions generating myofibroblasts promote the cancer progression. The stromal extracellular matrix (ECM) contains several molecules such as collagen, elastin, fibronectin, hyaluronic acid, proteoglycans, glycoproteins etc. The cancer stroma presents several similarities with skin healing (108, 109). In cancers, stromal cells become over-activated, proliferate, and upregulate the ECM production. ECM protects growth factors from degradation and can modulate the activity of growth factors and cytokines (109–111). The main factors are the fibroblast growth factor, platelet-derived growth factor, tumor necrosis factor, epidermal growth factor, hepatocyte growth factor, and vascular endothelial growth factor.

Cancer cells release microparticles derived from the plasma which contribute to activate tumor-associated myofibroblasts. Cancer exosomes differentiate fibroblasts into myofibroblasts (112). In cancers, hyperpermeable vessels allow leakage of proteins from the plasma, fibrinogen and fibronectin into the stroma. Myofibroblasts derive from different precursor cells. EMT represents also a mechanism of myofibroblast generation during tumor development (113). In the tumor stroma, pericytes could acquire contractile myofibroblast properties (114, 115). Bone-marrow (BM)-derived MSCs and blood-circulating fibrocytes could be also sources for cancer-associated myofibroblasts. BM-derived cells can differentiate into myofibroblasts in tumor development (116, 117). MSCs target to the stroma environment of epithelial cancers (115, 118–121). In tumor-conditioned medium, BM-MSCs differentiate into myofibroblasts containing α-SMA (122).

With mechanical stress, TGF-β1, the most important myofibrogenic growth factor, plays a central role in myofibroblast activation and by upregulating the canonical WNT signaling (Figures 2, 3). In all circumstances in which TGF-β1 is upregulated, the canonical WNT pathway is overactivated. TGF-β1 is involved in tumor formation (123) and development (124–126). TGF-β1 induces ECM deposition and acts in various epithelial-mesenchymal interactions (127). Latency and activation of TGF-β1 is regulated by the latent TGF-β1 binding protein-1 (LTBP-1) (128, 129). In cancers, cells induce proangiogenic effects and favor EMT (124–126). TGF-β1 disrupts cell-cell junctions between vascular endothelial cells and favors extravasation of metastasizing cancer cells (130). In tumor cells, the TGF-β1 blockade decreases metastasis (131, 132). In the activated tumor stroma, myofibroblasts stimulate the synthesis and activation of TGF-β1 and generate an autocrine forward loop (133).

TGF-β1 is synthesized together with the latency-associated peptide (LAP) that is cleaved in the cell and linked to TGF-β1. This leads to the small latent complex (SLC) (134). In compliant low contractile proto-myofibroblasts, TGF-β1, and LAP form the small latent complex (SLC) (14, 134). Myofibroblasts secrete SLC which is covalently bound with LTBP-1 (134, 135). LTBP-1 belongs to the family of the fibrillin proteins and stores the latent TGF-β1 in the ECM. Integrins activate the latent TGF-β1 (136), which contains RGD for integrin binding in LAP (137–139). These mechanisms involve several αv integrins (140–142). Myofibroblasts exert a tension on the ECM-stored latent TGF-β1 through integrins. Traction force exerted on SLC induces a conformational change in LAP that releases activated TGF-β1. Stiff ECM favors differentiation into a myofibroblast phenotype (13) by activating the latent TGF-β1. Tumor-associated stroma is stiffer than the surrounding normal soft connective tissue (143–147). Activated cancer-associated fibroblasts produce several growth factors and cytokines that promote tumor progression such as FGF, EGF, TGF-β1, HGF, VEGF, stroma-derived factor-1, and the pro-inflammatory cytokines IL-1, IL-6, IL-8, and CXCL14 (148–150). They also provide conditions leading to the tumor progression (147). Increased stiffness of cancer-associated myofibroblasts modifies epithelial cells toward an invasive and proliferative phenotype.

## Canonical WNT/β-Catenin Signaling, Warburg Glycolysis, and Cancer

Upregulation of the canonical WNT pathway plays a pivotal role in metabolism and particularly in the aerobic glycolysis (Figure 6). The Warburg effect (15) denotes that cancer cells, even under aerobic conditions, favor the cytosolic glycolysis pathway rather than mitochondrial phosphorylation. The last product of cytosolic glycolysis is pyruvate, which is then converted into lactate, hence the term “aerobic glycolysis” given to this phenomenon. In terms of ATP production, aerobic glycolysis is much less efficient than mitochondrial oxidative phosphorylation due to the shunt of the tricarboxylic acid (TCA) cycle. In the aerobic glycolysis, one glucose molecule leads to the formation of only 2 ATP molecules, instead of 38 ATP molecules in mitochondrial oxidative phosphorylation. In cancer development, the role of the canonical WNT pathway in metabolism is now better understood (151, 152). Activation of the canonical WNT signaling via TCF/ LEF induces an activation of the aerobic glycolysis, through upregulation of glycolytic enzymes such as pyruvate dehydrogenase kinase 1 (PDK1), pyruvate kinase M2 (PKM2), lactate dehydrogenase A (LDH-A), monocarboxylate transporter 1 (MCT-1), and glucose transporter (Glut). Activation of PDK1 is required for the Warburg aerobic glycolysis. PDK1 phosphorylates the pyruvate dehydrogenase complex (PDH) which is then inhibited and therefore prevents the conversion of pyruvate into acetyl-CoA in mitochondria, blocking the pyruvate access to oxidative phosphorylation (153). In this manner, the WNT-driven Warburg metabolism favors the use of glucose for cancer cell proliferation (22). Upregulation of the canonical WNT pathway leads to β-catenin translocation in the nucleus where β-catenin interacts with TCF/LEF. This results in activation of the β-catenin target genes (PDK1, MTC-1, cMYC, CYCLIN D1, COX- 2, AXIN- 2) (7, 8, 21, 22) (Figures 2, 3, 6). Upregulation of the canonical WNT pathway leads to cell proliferation, EMT, migration and angiogenesis (11, 20, 154–156). In various cancers, PDK-1 is upregulated (22, 157–159), so that the pyruvate conversion into acetyl-CoA in mitochondria is decreased and the WNT-driven Warburg aerobic glycolysis induced in spite of the availability of oxygen. In the cytosol, pyruvate is converted into lactate through LDH-A activation. Upregulation of both LDH-A and MCT-1 leads to lactate formation and the secretion of lactate outside of the cell. This induces vascularization (160) and anabolic production of biomass for nucleotide synthesis (161, 162). Furthermore, PDK-1 and−2 create conditions conductive to angiogenesis (163, 164). Inhibition of the canonical WNT signaling decreases the PDK-1 level via the transcription regulation and reduces *in vivo* tumor growth (22). cMYC, a target gene of β-catenin activates the aerobic glycolysis and glutaminolysis, induces the uptake of glutamine into the cell and mitochondria, activates LDH-A and activates aspartate synthesis that finally leads to nucleotide synthesis (165, 166). cMYC also stimulates the hypoxia-inducible factor-α (HIF-1α) which in turn regulates PDK-1 (167). In carcinogenesis, HIF-1α activates the Warburg aerobic glycolysis (168). In this process, a part of the pyruvate is converted into acetyl-Co-A which enters the TCA cycle, and is converted into citrate. This leads to the synthesis of proteins and lipids. Cellular accumulation of metabolic intermediates such as glycine, aspartate, serine, and ribose, allows *de novo* synthesis of nucleotides (Figure 6), contributing to cell growth and proliferation. Lactate also induces angiogenesis. Importantly, aerobic glycolysis is also induced in response to TGF-β1 (169) and glucose consumption is increased in cancer cells. High glucose concentration regulates tumor-related processes. Glucose itself directly influences the canonical WNT signaling (170). High glucose levels enhance the nuclear translocation of β-catenin in response to canonical WNT activation. In cancer cells, glucose-induced β-catenin acetylation increases canonical WNT signaling. Stimulation of the canonical WNT pathway leads to activation of HIF-1α causing metabolic remodeling (154, 171) and accentuates the Warburg effect. Thus, cancer cells use the Warburg effect at all oxygen levels (172). The increase in lactate production and the activation of HIF-1α by the upregulated canonical WNT signaling are associated with the increase of angiogenesis and poor prognosis of cancers (173). Lactate released from cancer cells, via the MCT-1 transporter allows entry of lactate anion into cancer endothelial cells. In normoxic endothelial cells, lactate activates HIF-1α in a positive feedback loop by blocking HIF-1α prolyl hydroxylation and then prevents HIF labeling by the von Hippel-Lindau protein (163, 173, 174).

Lactate released from the cell initiates a transformation of the microenvironment independently of hypoxia. This enables angiogenesis and activation of the NF-kappaB pathway and prevents cell death through antiapoptotic proteins facilitating survival of cancer cells in a hostile environment (175). Knockdown of PDK-1 reverts the Warburg phenotype, diminishes the normoxic HIF-1α expression, hypoxic cell survival, invasion, and inhibits cancer development. Glycolysis metabolites due to high PDK-1 expression may promote HIF-1α activation. This sustains a feed-forward loop for cancer development. If corrected, these metabolic abnormalities may represent new cancer therapies which can synergize with other cancer treatments.

### PI3K-AKT Pathway in WNT-Driven Aerobic Glycolysis and Cancer

The serine/threonine kinase AKT or protein kinase B (PKB) is often stimulated in human cancers (176). AKT activation constitutes a hallmark of the Warburg effect in most cancers. In tumor cells, hyperactivation of phosphatidyl-inositol 3-kinase (PI3K)-protein kinase B (AKT) signaling is associated with an augmented rate of glucose metabolism. AKT is activated by PIP3. PI3K synthesizes phosphatidylinositol-3,4,5-triphosphate (PIP3) from PIP2. The PI3K-AKT signaling induces cell growth and proliferation, cell migration, and angiogenesis. This signaling is induced by the linking of extracellular ligands to a receptor tyrosine kinase (RTK) in the plasma membrane (Figure 6) and is upregulated in several cancers. The AKT signaling activates the canonical WNT pathway by decreasing the GSK-3β activity (inactive form: pGSK-3β). AKT activation increases the Warburg aerobic glycolysis in cancer. AKT increases the ATP production and enhances glycolytic metabolism by several mechanisms. It increases glucose uptake by increasing the Glu transporter expression. AKT activates mTORC1, which promotes HIF-1α under normoxic conditions and increases LDH activity. The PI3K-AKT signaling increases the hypoxia-inducible transcription factor (177). AKT phosphorylates and stimulates phosphofructokinase-2 (PFK2), which in turn induces activation of the phosphofructokinase-1 (PFK1) by the PFK2 product fructose-2,6-bisphosphate (Fru-2,6-P2), an activator of PFK1. PFK1 is an allosteric enzyme responsible for glycolytic oscillations and can lead to instability beyond which a new state can be created in time and in space. These oscillations are dissipative structures initially described by Goldbeter and Lefever (178) and behave far-from-equilibrium (60, 179, 180). Increase in activity of PFK-1 is characteristic of tumor cells and is induced by oncogenes.

Upregulation of the PI3K-AKT signaling is frequently associated with cancers, such as glioblastomas, breast, pancreatic, and ovarian cancers (181). AKT mRNA is increased in breast and prostate cancers. PI3K-AKT induces angiogenesis by activating VEGF in endothelial cells and acting on the endothelial nitric oxide synthase. This stimulates vasodilation and vascular remodeling (182). The PI3K-AKT pathway is inhibited by the phosphatase and tensin homolog (PTEN) (183) (Figure 6). PTEN is a PIP3-phosphatase and inhibits PI3K. As PI3K-AKT is a key signaling activated in cancer, PTEN appears to be an interesting target against the tumor progression.

### Reverse Warburg Effect

The Warburg effect does not take into account the metabolic interactions between cancer cells and the microenvironment (184, 185). The reverse Warburg effect (186) is a new metabolic process describing interacting between cancer cells and neighboring cancer-associated fibroblasts (CAFs) or stroma cells (187). In several human tumor cells, aerobic glycolysis contributes <50% for energy production (188–191) and some cancer cells exhibit high rates of the mitochondrial oxidative phosphorylation system (OXPHOs) (192–194). Thus, OXPHOs and aerobic glycolysis are not always mutually exclusive. Heterogeneous cancer cells can show various metabolic phenotypes (195–198). The OXPHOs metabolic pathway does not invalidate the Warburg effect but rather exhibits the complex plasticity of cancer metabolism and is involved in proliferation, metastasis, angiogenesis, and drug resistance in cancer cells. Oxidative stress in the two-compartment metabolic coupling and variations in cellular electromagnetic field could initiate the reverse Warburg effect. Cancer cells secrete ROSs in the microenvironment, which induces oxidative stress in CAFs which is strongly correlated to the reverse Warburg effect. Oxidative stress mechanism drives CAFs-cancer cells metabolic coupling and favors tumor growth via genetic and metabolic mechanisms. Oxidative stress related loss of stromal caveolin-1 (Cav-1) increases the production of ROSs in tumor cells, which initiates the cascade of oxidative stress in CAFs via a positive feedback mechanism (187, 199, 200) and plays a role in cancer proliferation, recurrence and prognosis (201, 202). Although the reverse Warburg effect and the role of the canonical WNT signaling on the reverse Warburg effect are not totally understood to-day, in the future they could represent a new approach for cancer treatment.

## Canonical WNT Signaling, Circadian Rhythms (CRs), and Cancer

CRs are endogenous rhythms that last ~24 h, due to a negative feedback produced by a protein acting on the expression of its own gene (6, 203, 204). In mammals, CRs involve several major factors such as brain and muscle aryl-hydrocarbon receptor nuclear translocator-like1 (BMAL1), circadian locomotor output cycles kaput (CLOCK), period 1 (PER1), period 2 (PER2), and period 3 (PER3) (205, 206). CRs are involved in hormone secretion, heart rate and blood pressure, sleep-awake, feeding patterns, energy metabolism, and body temperature. Importantly, disruption of CRs appears linked to cancer, leading to aberrant cellular proliferation and involving the canonical WNT signaling (23). Due to several connections between cellular metabolism and the circadian clock, abnormal metabolism described in cancers may be a consequence of disrupted CRs (207). A decreased BMAL1 activity changes the behavior of genes involved in the canonical WNT pathway (208). β-catenin induces PER2 degradation modifying the circadian clock gene expression in intestinal mucosa of ApcMin/+ mice (209). A deceased expression of PER1 and/or PER2 has been reported in numerous cancers: breast tumor (210), prostate tumor (211), pancreatic tumor (212), colorectal tumor (213), chronic myeloid leukemia (214), glioma (215, 216), and intestinal epithelial neoplastic transformation (209).

From a thermodynamic viewpoint, CRs operate far from equilibrium and are dissipative structures (56, 60, 217). By applying Schrödinger's concepts to the modern thermodynamics of physical, chemical and biological systems that behave far from equilibrium, Prigogine and his colleagues opened a new understanding for the description of dissipative structures which occupy a key role in the world of living beings. Cancers are exergonic processes in which heat flows from the cancer to its surroundings (218). In cancer cells, the entropy production rate increases and characterizes irreversible processes driven by changes in thermodynamics properties (61).

## Canonical WNT Signaling, Inflammation, Fibrosis, and Cancer

### Inflammation

Chronic inflammation contributes to cancer development (219, 220). The role of prostaglandin E2 (PGE2) by stimulating the canonical WNT pathway has been well-established. The crosstalk between PGE2 and the canonical WNT signaling indicates that chronic inflammation caused by a prolonged increase of PGE2 may upregulate the canonical WNT signaling leading to cell proliferation and cancer. PGE2 favors the transcription of β-catenin (221, 222) and leads to growth of cancer cells such as in colon cancer. Non-steroidal anti-inflammatory drugs (NSAIDs) improve the treatment of colorectal cancer (223), partly due to interference with the β-catenin signaling and by inhibiting the PGE2 synthesis. PGE2 regulates the canonical WNT activity of hematopoietic stem cells (HSCs) in the zebrafish. Inhibition of PGE2 synthesis blocks the WNT-induced alterations in HSCs. PGE2 alters the β-catenin degradation via the cAMP/PKA signaling. In stem cells, WNT upregulation requires PGE2 (224). Dimethyl-prostaglandin E2 increases HSCs *in vivo* and increases the canonical WNT pathway activity (225). Conversely the canonical WNT signaling activates interleukins (IL)-7R and IL-2Rβ. In neuroectodermal stem cells, PGE2 interacts with the canonical WNT pathway partly through PKA and PI3K (226). PGE2 upregulates several WNT-target genes such as Ctnnb1, Ptgs2, Ccnd1, Mmp9.

### Cancer and Fibrosis: A Similar Pathway

Several intracellular processes involved in cancers are similar to those encountered in fibrosis, and particularly an activation of the canonical WNT /TGF-β1 signaling (227). Similarly to that observed in cancer stroma, activation of this pathway leads to differentiation of fibroblasts into myofibroblasts. In cancer (mammary carcinoma, epithelial cells in tumor mammary glands), fibrotic lesions (nodules of Dupuytrens, hypertrophic scars) (228), and normal placental stem villi (229), the main myosin molecular motor in myofibroblasts is the non-muscle myosin IIA (NMMIIA). Contractile NMMIIA kinetics are particularly slow (230) and their entropy production rate is dramatically low (231). These contractile myofibroblasts allow skin retraction in wound healing (232–234). Patients suffering of cancer often receive a therapy by external ionizing radiations that leads to damages in both tumor cells and health tissues. Radiations induce numerous skin changes including inflammation and late radiation-induced fibrosis (RIF). RIF are characterized by fibroblast proliferation and myofibroblast differentiation, DNA damage, inflammation by stimulation of the NF-kappaB pathway (32). The over-expression of TGF-β1 and the upregulated canonical WNT signaling are involved in the molecular mechanism underlying the RIF.

## Conclusions and Perspectives

It is currently well-demonstrated that the canonical WNT signaling intervenes at every stages in development of many cancers. When activated, this signaling induces and initiates several periodic or oscillating phenomena, such as the cell division cycle, the immune cycle, numerous circadian rhythms, and phosphofructokinase oscillations that are dissipative structures. This is because β-catenin modifies the expression of a considerable number of genes involved in one or others of these decisive areas of the cancer pathogeny. Thus, CYCLIN D1 and cMYC influence the cell division cycle. TGF-β1 also plays an important role activating the canonical WNT signaling directly or indirectly, and by acting on both the PI3K-AKT and GSK-3β pathways. Activation of the canonical WNT signaling affects many enzymes or transporters, resulting in Warburg glycolysis, which diverts pyruvate metabolism to lactate, a useful pathway for protein synthesis in tumor cells. All these different functions of the canonical WNT give it an eminent status as target immunotherapies against cancer.

## Author Contributions

All authors listed have made a substantial, direct and intellectual contribution to the work, and approved it for publication.

### Conflict of Interest

The authors declare that the research was conducted in the absence of any commercial or financial relationships that could be construed as a potential conflict of interest.

## Abbreviations

APC: adenomatous polyposis coli
AEC: alveolar epithelial cells
BMAL1: circadian brain and muscle aryl-hydrocarbon receptor nuclear translocator-like1
CR: circadian rhythm
CTGF: connective tissue growth factor
CTLA-4: cytotoxic T lymphocyte-associated antigen-4
DCs: Dendritic Cells
DKK1: dickkopf-1
DSH: disheveled
EGF: epidermal growth factor
EMT: epithelial-mesenchymal transition
FGF: fibroblast growth factor
FZD: Frizzled
Glu: glucose transporter
GSK-3β: glycogen synthase kinase-3β
HGF: hepatocyte growth factor
HIF: hypoxia-inducible transcription factor
IRF8: interferon regulatory factor 8
LDH: lactate dehydrogenase
LAP: latency-associated peptide
LTBP-1: latent TGF-β1 binding protein-1
CLOCK: locomotor output cycles kaput
LRP5/6: low-density lipoprotein receptor-related protein 5/6
MCT-1: monocarboxylate lactate transporter-1
NMMIIA: non-muscle myosin IIA
PER: period
PPARγ: peroxisome proliferator-activated receptor gamma
PI3K: phosphatidyl-inositol 3-kinase
PDGF: platelet-derived growth factor
PTEN: phosphatase and tensin homolog
PFK: phosphofructokinase
AKT: protein kinase B
PD-1: programmed cell death protein1
PDH: pyruvate dehydrogenase complex
PDK: pyruvate dehydrogenase kinase
PKM2: pyruvate kinase M2
RTK: receptor tyrosine kinase
Treg cell: Regulatory T cell
SLC: small latent complex
SMA: smooth muscle actin
ECM: extracellular matrix
TCF/LEF: T-cell factor/lymphoid enhancer factor
TGF: Transforming Growth Factor
Th: T helper
TCA: tricarboxylic acid
TNF-β: tumor necrosis factor beta
VEGF: vascular endothelial growth factor.
